# Molecular mechanisms of normal and pathological aging

**DOI:** 10.1186/1750-1172-10-S2-O1

**Published:** 2015-11-11

**Authors:** Carlos López-Otín

**Affiliations:** 1 Departamento de Bioquímica y Biología Molecular, IUOPA, Universidad de Oviedo, Oviedo, Spain

## 

We have recently defined nine molecular and cellular hallmarks that represent common denominators of aging in different organisms. These hallmarks are: genomic instability, telomere attrition, epigenetic alterations, loss of proteostasis, deregulated nutrient-sensing, mitochondrial dysfunction, cellular senescence, stem cell exhaustion, and altered intercellular communication. On the other hand, parallel studies of our laboratory on accelerated aging syndromes, including Hutchinson-Gilford Progeria Syndrome (HGPS) and Nestor-Guillermo Progeria Syndrome (NGPS), have provided relevant information about these hallmarks of aging. HGPS is caused by a point mutation in the *LMNA* gene that yields a truncated form of prelamin A called progerin, which is also produced during normal aging. Over the last years, the generation of mouse models of HGPS and other progeroid laminopathies has shed light on the molecular alterations functionally involved in these diseases. Thus, *knock-out mice* deficient in Zmpste24 metalloproteinase implicated in prelamin A maturation, *mosaic mice* containing *Zmpste24*-deficient and *Zmpste24*-proficient cells, and *knock-in mice* carrying the human HGPS mutation which causes progerin accumulation, have allowed us to demonstrate that progeroid laminopathies result from the combined action of both cell-autonomous and systemic factors. Accordingly, we have shown that nuclear envelope defects causative of these complex diseases lead to alterations in stem cell functionality, epigenetic abnormalities, perturbations in cell senescence pathways, metabolic changes and chronic activation of inflammatory responses. We have also demonstrated that the genetic or pharmacological blockade of these altered pathways prevents the development of many age-associated features of these progeroid mice and extends their longevity. On this basis, we have developed therapeutic strategies for progeroid laminopathies which are now in clinical trials coordinated by Pr. Nicolas Lévy for the treatment of HGPS patients. These findings illustrate the importance of mouse models for designing therapeutic strategies to treat rare and dramatic progeroid syndromes as well as for improving our knowledge of the universal and complex process of human aging.

